# ABHD17C regulates the efficacy of lenvatinib in suppressing hepatocellular carcinoma

**DOI:** 10.1080/15384047.2026.2693350

**Published:** 2026-06-25

**Authors:** Linpei Wang, Jiawei Wang, Xiaoqiu Ma, Jinfa Huang, Yingming Sun, Chunfeng Shi, Jiahui Zhang, Peng Li, Hao Xu, Hongtan Lin, Wei Wang

**Affiliations:** a Department of Hepatobiliary and Pancreatic Surgery, The Second Affiliated Hospital of Fujian Medical University, Quanzhou, Fujian, China; b Department of Health Medicine, The 910th Hospital of People's Liberation Army, Quanzhou, Fujian, China; c Department of Oncology and Hematology, Shishi Hospital, Quanzhou, Fujian, China; d Department of Medical and Radiation Oncology, Sanming First Hospital Affiliated to Fujian Medical University, Sanming, Fujian, China; e Department of Hepatobiliary Surgery, The First Affiliated Hospital of Zhejiang Chinese Medical University, Zhejiang Provincial Hospital of Chinese Medicine, Hangzhou, Zhejiang, China; f The First School of Clinical Medicine, Zhejiang Chinese Medical University, Hangzhou, Zhejiang, China; g Department of Surgical Oncology, Sanming First Hospital Affiliated to Fujian Medical University, Sanming, Fujian, China

**Keywords:** Hepatocellular carcinoma, lenvatinib, ABHD17C, organoid, single-cell sequencing

## Abstract

**Introduction:**

Lenvatinib is a first-line therapy for hepatocellular carcinoma (HCC), but its clinical efficacy is limited by drug resistance. ABHD17C, a depalmitoylation enzyme involved in HCC progression, has not been investigated in lenvatinib response. This study aimed to determine whether ABHD17C regulates the anti-tumor efficacy of lenvatinib in HCC.

**Methods:**

Published single-cell RNA sequencing (scRNA-seq) data were analyzed to characterize ABHD17C expression in the HCC tumor microenvironment. Functional assays were performed in HCC cell lines to evaluate the effects of lenvatinib and ABHD17C modulation. The findings were validated using HCC xenograft mouse models and patient-derived tumor organoids.

**Results:**

scRNA-seq analysis showed that ABHD17C is associated with an immunosuppressive tumor microenvironment characterized by reduced CD8⁺ T cell infiltration, increased T cell exhaustion, and abnormal intercellular communication. In vitro, lenvatinib inhibited proliferation, migration, and invasion while inducing apoptosis and cell cycle arrest in HCC cells. These effects were significantly attenuated by ABHD17C overexpression but enhanced by ABHD17C depletion. In vivo, ABHD17C-overexpressing xenografts were less responsive to lenvatinib, exhibiting increased tumor growth and reduced apoptosis. Similarly, in patient-derived organoids, ABHD17C overexpression diminished lenvatinib efficacy. Notably, lenvatinib reduced ABHD17C expression in organoids, suggesting potential feedback regulation.

**Conclusion:**

ABHD17C promotes an immunosuppressive tumor microenvironment and attenuates the anti-tumor effects of lenvatinib in HCC. Targeting ABHD17C may represent a potential strategy to enhance lenvatinib sensitivity and improve therapeutic outcomes.

## Introduction

Lenvatinib is an inhibitor of multiple kinases that acts on several receptors, including VEGFRs, FGFRs, PDGFRα, RET, and KIT.^1^ These receptors are essential for regulating tumor angiogenesis, reducing the blood supply to the tumor, cell growth, and progression,^2^ making lenvatinib a valuable therapeutic agent for several cancers, such as renal cell carcinoma, thyroid cancer, and hepatocellular carcinoma (HCC).^3-5^ However, its clinical efficacy is often hindered by drug resistance, which may arise from factors such as epigenetic alterations and the tumor microenvironment.^6-8^ Despite these findings, the precise mechanisms driving lenvatinib resistance in HCC remain incompletely understood.

S-palmitoylation, a crucial post-translational modification of proteins, covalently attaches a 16-carbon saturated fatty acid to target proteins on their cysteine residues.^9^ This process is vital for adjusting and controlling protein localization, stability and function.^10-13^ The ABHD17 family of enzymes, comprising ABHD17A, ABHD17B, and ABHD17C, plays a critical role in reversing this modification by removing substrates' S-palmitoylation. These enzymes exhibit high sequence similarity and are ubiquitously expressed in diverse cell types.^14^ ^,^ ^15^

Given the importance of protein palmitoylation in cancer biology,^16^ ^,^ ^17^ the ABHD17 family has emerged as a significant player in tumor development. For example, ABHD17 enzymes can promote cancer progression by depalmitoylating oncogenic RAS proteins, enabling their release from the cell membrane and subsequent activation of downstream signaling pathways.^14^ ^,^ ^15^ Recent studies from our group have demonstrated that ABHD17C is overexpressed in HCC and functions as an oncogene, driving malignant behaviors and modulating the PI3K/AKT pathway.^18^ ^,^ ^19^ Nevertheless, the role of ABHD17C in modulating drug sensitivity in HCC and shaping the tumor microenvironment remains inadequately elucidated.

Here, we first analyzed the potential relationship between ABHD17C expression and the characteristics of the HCC tumor microenvironment using scRNA-seq data. Our analysis revealed that ABHD17C is associated with an immunosuppressive tumor microenvironment characterized by reduced CD8⁺ T cell infiltration, increased T cell exhaustion, and impaired intercellular communication. We then examined the influence of ABHD17C on the anti-tumor effects of lenvatinib on HCC. Our results revealed that lenvatinib treatment downregulates ABHD17C expression in HCC cells. Excessive ABHD17C attenuated the inhibitory effects of lenvatinib on cell viability, cell cycle progression, migration, invasion, and its capacity to induce apoptosis. These findings were further confirmed in an HCC xenograft mouse model and in tumor organoids derived from primary HCC tissues, underscoring the potential role of ABHD17C in driving lenvatinib sensitivity.

## Methods and materials

### Published single-cell RNA sequencing (scRNA-seq) dataset analysis

The expression matrix of filtered read counts from the human HCC scRNA-seq dataset GSE149614 was downloaded from the Gene Expression Omnibus and processed using the Seurat (v5.3.0) R package. The raw counts were log-normalized using the *LogNormalize* method with a scale factor of 10,000. The top 2000 highly variable genes were identified using the variance stabilizing transformation (VST) method, followed by data scaling with *ScaleData()*. Principal component analysis (PCA) was performed on the normalized data, and the first 20 principal components were used for downstream integration and dimensionality reduction. Cross-sample integration was conducted using canonical correlation analysis (CCA). K-nearest neighbor (KNN) graphs were constructed using *FindNeighbors()* (dims = 1:20), and clustering was performed with *FindClusters()* (resolution = 1) to identify cell subpopulations. The results were visualized using Uniform Manifold Approximation and Projection (UMAP) via *RunUMAP()* (dims = 1:20).

Hepatocyte malignancy in HCC samples was inferred using the CopyKAT (v1.1.0) R package to estimate copy number variations, with T and NK cells serving as diploid references. Aneuploid cells identified by CopyKAT were classified as malignant. Cell‒cell communication analysis was performed using CellChat (v1.6.1).

### Cell culture and plasmids

All cell lines, including HEK293T cells (#IM-H222), PLC/PRF/5 (#IM-H413), and Huh7 (#IM-H040), were acquired from Immocell and cultured in DMEM with 10% fetal bovine serum (FBS) at 37 °C in a 5% CO_2_ incubator, and the cells were supplied with authentication via short tandem repeat (STR) profiling and confirmed to be free of mycoplasma contamination. To construct the plasmid for ABHD17C overexpression (OE), the coding sequence of ABHD17C was inserted into the pcDNA3.3 backbone. To deplete ABHD17C, specific shRNAs were inserted into the pLKO.1 backbone, producing the shABHD17C-1, shABHD17C-2, and shABHD17C-3 plasmids. A non-specific shRNA (shNC) was used as a negative control. Plasmid transfection was conducted using Lipofectamine 2000 (Invitrogen, #11668019). The primers are as follows: pcDNA3.3-ABHD17C-F: 5′-CTAGAGAATTCGGATCCATGCCCGAGCCAGGCCCCAGGATGAAC-3′,

pcDNA3.3-ABHD17C-R: 5′-AGCTTCCATGGCTCGAGGGAATTAGGAAGTTCGTGAG-3′; ABHD17C-sh1-F: 5′-CCGGGCGTGAGTCCCGAGAACATTACTCGAGTAATGTTCTCGGGACTCACGCTTTTT-3′, ABHD17C-sh1-R: 5′- AATTAAAAAGCGTGAGTCCCGAGAACATTACTCGAGTAATGTTCTCGGGACTCACGC-3′; ABHD17C-sh2-F: 5′- CCGGTATGAATGCGCAGCGGTAATTCTCGAGAATTACCGCTGCGCATTCATATTTTT-3′, ABHD17C-sh2-R: 5′- AATTAAAAATATGAATGCGCAGCGGTAATTCTCGAGAATTACCGCTGCGCATTCATA-3′; ABHD17C-sh3-F: 5′- CCGGCATCAACTGTAACCATATAAACTCGAGTTTATATGGTTACAGTTGATGTTTTT-3′, ABHD17C-sh3-R: 5′- AATTAAAAACATCAACTGTAACCATATAAACTCGAGTTTATATGGTTACAGTTGATG-3′; shNC-F: 5′- CCGGTTCTCCGAACGTGTCACGTTTCTCGAGAAACGTGACACGTTCGGAGAATTTTT-3′, shNC-R: 5′- AATTAAAAATTCTCCGAACGTGTCACGTTTCTCGAGAAACGTGACACGTTCGGAGAA-3′.

### qPCR

Total RNA was extracted using RNA Isolater Total RNA Extraction Reagent (Vazyme, R401-01). Reverse transcription and subsequent qPCR were performed with HiScript II Q RT SuperMix for qPCR (Vazyme, R223-01) and ChamQ SYBR Color qPCR Master Mix (Vazyme, Q311-01), respectively. The qPCR machine was a LightCycler 96 (Roche, USA). The 2^−ΔΔCt^ method was applied to calculate the relative expression of target genes. The primers are as follows: ABHD17C-qF: 5′-ATGTCTGGTTTGCGTGTG-3′, ABHD17C-qR: 5′-CTTGTCAATGCTGGGGAAA-3’; 18S rRNA-qF: 5′-CGACGACCCATTCGAACGTCT-3′, 18S rRNA-qR: 5′-CTCTCCGGAATCGAACCCTGA-3′.

### Western blot

Total protein was prepared with the use of RIPA lysis buffer supplemented with protease inhibitors (Beyotime Biotechnology, P1006). The lysates were centrifuged at 13,000 rpm for 15 min to obtain the supernatant. The BCA kit (TIANGEN, PA115) was applied to measure the total protein concentration. 10 μg of denatured protein from each sample was separated by 10% SDS‒PAGE, transferred onto PVDF membranes, and then blocked with 5% nonfat milk for 1 h. Following blocking, the membranes were incubated with antibodies overnight. After TBST washes, the membranes were incubated with secondary antibody solutions for 2 h. The membranes were rinsed 3 more times with TBST, and protein signals were visualized by ECL working solution (Beyotime Biotechnology, P0018M). The chemiluminescent signals were captured using X-ray films, and the band intensities were quantified by ImageJ. The antibodies used are listed in Table 1.

**Table 1.** Antibody used for Western blot.

Target	Producer	No.	Dilution
ABHD17C	ORIGENE, China	TA335493	1:500
Cleaved-caspase 3	Cell Signaling Technology, USA	#9661	1:500
p-PI3K		#4228	1:500
Bcl2		#2876	1:500
Pro-caspase 3	Abcam, UK	Ab32150	1:500
BAX	Immunoway, USA	YM3619	1:500
PI3K	Proteintech, China	20584-1-AP	1:500
p-AKT		80455-1-RR	1:500
AKT		60203-2-Ig	1:500
Beta-Actin		20536-1-AP	1:1000
VEGFR		26415-1-AP	1:500
HRP-anti-rabbit IgG		SA00001-2	1:10000
HRP-anti-mouse IgG		SA00001-1	1:10000

### Methyl thiazolyl tetrazolium (MTT) assay

MTT (Yeasen, 40201ES80) was utilized to assess cell viability. In short, cells were inoculated into 96-well plates at a density of 5000 cells in each well, left to attach overnight, then treated with varying concentrations of lenvatinib (Selleck, S1164) and incubated for 72 h. After treatment, 10 µL of MTT solution was added, and incubated for 4 h. The medium was removed, 150 µL of DMSO was added, and the OD_490_ values were measured. The IC_50_ value, defined as the concentration of lenvatinib required to inhibit 50% of cell viability, was calculated.

### Cell counting kit-8 (CCK-8) assay

Prepare a single-cell suspension with culture medium containing 10% FBS. Seed cells into a 96-well plate at a density of 2000 cells per well in a total volume of 100 μL. Incubate at 37 °C with 5% CO₂ for 48 h. Add 10 μL of CCK-8 solution (Beyotime, C0037) to each well, and continue incubation for 1 h. Measure the absorbance value of each well at a wavelength of 450 nm using a microplate reader, and calculate cell viability.

### Colony formation assay

700 cells in each well were inoculated into 6-well plates and cultured for 12 d. Afterward, the cells were fixed with 4% paraformaldehyde (Beyotime Biotechnology, P0099) for 30 min and rinsed 3 times with PBS. The colonies were then stained with 0.5% crystal violet solution for 15 min, followed by another PBS rinse. The plates were then air-dried, and colonies were observed and imaged. Colony numbers were counted by ImageJ.

## 7-AAD and annexin V-FITC/PI staining assays

The cell cycle was analyzed using 7-AAD staining assays. Briefly, 5 × 10^6^ cells were harvested, fixed in ethanol overnight, and resuspended in PBS. Then, the cells were incubated with RNase A for 30 min at 37 °C. After washing, the cells were stained with 5 μL of 7-AAD solution (Bestbio, BB-41044) in PBS and incubated in a light-protective environment at 4 °C for 30 min. The cell cycle was detected by flow cytometry, and the data were processed with FlowJo.

Cell apoptosis was detected by an Annexin V-FITC/PI kit (Yeasen, 40302ES50). In short, 5 × 10^5^ cells were collected, washed with PBS, and resuspended in 100  μL of binding buffer. The cells were then stained with 5 μL of Annexin V-FITC and 5 μL of PI solution for 10 min in the dark. After adding 400 μL of binding buffer, the samples were analyzed by flow cytometry.

### Transwell assay

Cell migration and invasion ability were detected in 24-well Transwell chambers with 8 μm pores (Corning, #3422). For the detection of invasion ability, the upper chambers were pre-coated with 100 μL of Matrigel (Corning, #356235). The cells were resuspended in serum-free medium (3 × 10^5^ cells/mL), and 200 μL of the cell suspension was added to the upper chamber. The lower chamber was filled with 700 μL of complete medium. After 48 h of incubation, the cells were fixed with methanol, stained with 0.05% crystal violet solution (Solarbio, C8470, China), and imaged. The migration assay followed the same procedure, except that the upper chambers were not pre-coated with Matrigel.

### Wound healing assay

HCC cells (1 × 10^6^) were inoculated into 6-well plates and transfected with the indicated plasmids. After 24 h, a wound was created in each well using a pipette tip, and the cell debris was removed with PBS. Serum-free medium (4 mL) was added, and the wounds were imaged immediately (0 h) and after 24 h using an IX73 inverted microscope.

### Xenograft assay

BALB/c nude mice (4–6 weeks) were acquired from Vital River Ltd. and fed with food and water in free conditions; confounding factors were controlled, and blinding was employed. This study did not establish specific inclusion or exclusion criteria for the animals. Using a random number table to generate the randomization sequence, and the mice were randomly divided into 3 groups. For tumor induction, approximately 5 × 10^6^ control or ABHD17C-overexpressing Huh7 cells resuspended in 100 µL of PBS were subcutaneously injected into the right flank of each mouse. The mice were stochastically divided into treatment and control groups. The treatment group received lenvatinib (10 mg/kg/d) via daily oral gavage starting 14 d after tumor cell transplantation until sacrifice, while the control group received a vehicle solution. The animals were grouped as follows: Vector, Vector + Lenvatinib, and ABHD17C OE + Lenvatinib, with 6 mice in each group (*n* = 6). Tumor sizes were measured regularly using calipers, and the study was terminated when the tumors reached 1500 mm^3^ or after 21 d. The mice were sacrificed using CO_2_ overdose exposure for 1 min, according to ethical guidelines, and the tumors were excised.

### HCC organoid establishment

HCC tissues were acquired from The Second Affiliated Hospital of Fujian Medical University with approval from the Ethics Committee (No. 2023-FYFELL-470). Written informed consent was acquired from the patients. Organoid establishment and culture procedures were performed as described in a previous publication.^20^

### Immunohistochemistry (IHC) and immunofluorescence (IF)

For IHC, mouse xenograft tumor tissue or HCC organoids were fixed with 4% paraformaldehyde, embedded in paraffin, and sectioned into 4-μm slices. Deparaffinized sections were then rehydrated, followed by antigen retrieval using citrate buffer. Endogenous peroxidase activity was quenched, and incubated with 10% goat serum at 37 °C for 30 min. Then, the sections were incubated with an antibody targeting Ki-67 (ab15580, Abcam, 1: 500) overnight at 4 °C. The sections were then incubated with HRP-conjugated secondary antibodies. The signals were detected by DAB staining. After that, the sections were counterstained with hematoxylin, dehydrated, cleared, and mounted.

For IF, the HCC organoids were fixed with 4% paraformaldehyde, embedded in paraffin, and sectioned into 5-μm slices. Deparaffinized sections were then rehydrated, followed by antigen retrieval using EDTA 9.0. Endogenous peroxidase activity was quenched, and incubated with 5% BSA. The sections were incubated with antibodies targeting Ki-67 (#28074-1-AP, Proteintech, 1: 1500), AFP (#14550-1-AP, Proteintech, 1: 100), or GPC3 (#A23410, Abclonal, 1: 100) overnight at 4 °C. The sections were incubated with Alexa Fluor 555-labeled secondary antibodies and counterstained with DAPI-containing mounting medium. The signals were observed and imaged by a microscope.

To detect apoptosis in tumors or organoids, the TUNEL assay was conducted using kits from UElandy (#T6014L) or Servicebio (#G1507-100T).

### ATP level measurement

To determine the ATP level of the organoids, a kit from Beyotime Biotechnology (S0027) was used. The OD values were measured by Microplate Luminometer Orion II (Berthold Technologies GmbH, Germany).

### Statistical analysis

GraphPad Prism 8 was used for statistical analysis. *p* < 0.05 was considered statistically significant, and the specific statistical test used for *p*-value calculation is indicated in the figure legends. Before conducting parametric tests, the normality of the data was assessed to confirm a normal distribution. All the data are presented as the form of the mean ± the standard error of the mean (SEM). In the bar-dot plots, each data point represents one biological replicate with or without technical triplicates. All the data were independently replicated biologically three times, with MTT, qPCR, and ATP assays additionally conducted in technical triplicates.

## Results

### ABHD17C expression is associated with an immunosuppressive tumor microenvironment in HCC

**Figure 1.**
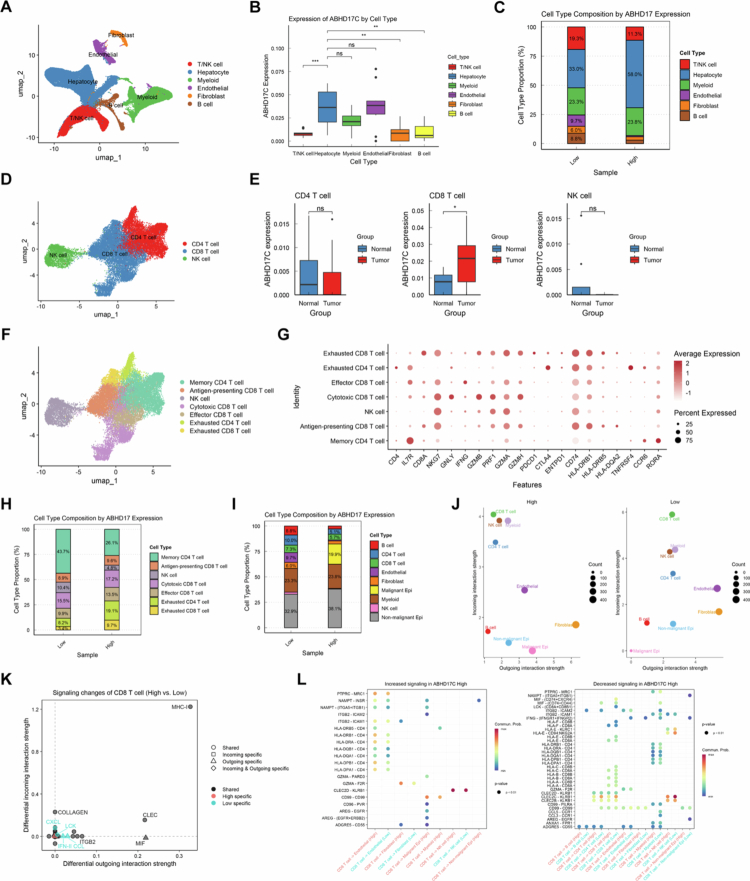
ABHD17C expression is associated with an immunosuppressive tumor microenvironment in HCC. (a) UMAP visualization of major cell populations in HCC and adjacent normal liver tissues from GSE149614. (b) Distribution of ABHD17C expression across major annotated cell types. (c) Comparison of major cell type proportions between the ABHD17C-high and ABHD17C-low tumor groups. (d) UMAP visualization of T/NK cell subclusters. (e) Box plots showing the expression profiling of ABHD17C in CD4^+^ T, CD8^+^ T, and NK cells. (f) UMAP visualization of higher resolution of T/NK cell subsclusters. (g) Dot plot showing the markers for T/NK cell subpopulation annotation. (h) T/NK cell composition between ABHD17C-high and ABHD17C-low groups. (i) Cell composition differences between ABHD17C-high and ABHD17C-low tumors across major cell types, including malignant and non-malignant hepatocytes. (j) CellChat analysis showing altered interaction strength among major cell populations in the ABHD17C-high versus ABHD17C-low groups. (k) Signaling changes in CD8^+^ T cells between the ABHD17C-high and ABHD17C-low groups. (l) Comparison of receptor‒ligand interaction activity between the ABHD17C-high and ABHD17C-low groups. *p < 0.05; **p < 0.01; ***p < 0.001; ns, not significant. Wilcoxon Rank Sum Test is used for statistical analysis in (b, e).

To investigate the expression pattern of ABHD17C in the HCC microenvironment, we analyzed the public scRNA-seq dataset GSE149614. Six major cell populations were annotated using canonical marker genes, including T/NK cells, B cells, myeloid cells, hepatocytes, endothelial cells, and fibroblasts (Figures 1A and S1A–F). Overall, these cell populations exhibited similar distribution patterns between tumor and adjacent normal liver tissues (Figure S1G). Notably, the proportion of T/NK cells was markedly lower in tumors compared with normal tissues, whereas the proportion of hepatocytes was substantially increased (Figure S1H).

Among all the cell populations, ABHD17C expression was relatively enriched in hepatocytes, myeloid cells, and T/NK cells compared with other cell compartments (Figure 1B). Differential expression analysis further showed that ABHD17C expression was significantly elevated in tumor-derived T/NK cells and fibroblasts, whereas no significant changes were observed in other cell types (Figure S1I).

To further evaluate the relationship between ABHD17C expression and the cellular composition, tumor samples were stratified into ABHD17C-high and ABHD17C-low groups. The ABHD17C-high group exhibited a marked reduction in T/NK cell abundance together with a corresponding increase in hepatocyte proportion, suggesting that ABHD17C expression is closely associated with the abundance of these two populations (Figure 1C). Interestingly, no significant difference in ABHD17C expression was observed between malignant and non-malignant hepatocytes based on inferred CNV profiles (Figure S2A–C).

Further subclustering of T/NK cells demonstrated that ABHD17C expression was particularly elevated in tumor-derived CD8⁺ T cells (Figure 1D and E). Among the CD8⁺ T-cell subsets, exhausted CD8⁺ T cells showed the most pronounced expansion in the ABHD17C-high group, accompanied by increased exhausted CD4⁺ T cells and reduced NK cell abundance (Figure 1F–H). These findings suggest that elevated ABHD17C expression is associated with T-cell dysfunction and impaired anti-tumor immunity.

Cell composition and intercellular communication analyses further support this immunosuppressive phenotype. Malignant hepatocytes were strongly enriched in the ABHD17C-high group, whereas CD8⁺ T cells, CD4⁺ T cells, NK cells, and B cells were significantly reduced (Figure 1I). CellChat analysis revealed a reduced interaction strength between CD8⁺ T cells and other cells, together with the loss of key anti-tumor signaling pathways, including CXCL, LCK/TCR, IFN-II, and CCL signaling, in the ABHD17C-high group (Figure 1J and K). In contrast, ligand–receptor interactions associated with immune evasion and tumor-promoting signaling, including CD96–PVR, AREG–EGFR, and ADGRE5–CD55, were enhanced, whereas HLA–CD8-mediated self-activation and interactions with NK and myeloid cells were diminished (Figure 1L). Collectively, these findings indicate that high ABHD17C expression is associated with an immunosuppressed, CD8⁺ T cell–exhausted tumor microenvironment in HCC.

### Lenvatinib suppresses the malignant traits of HCC cells

**Figure 2.**
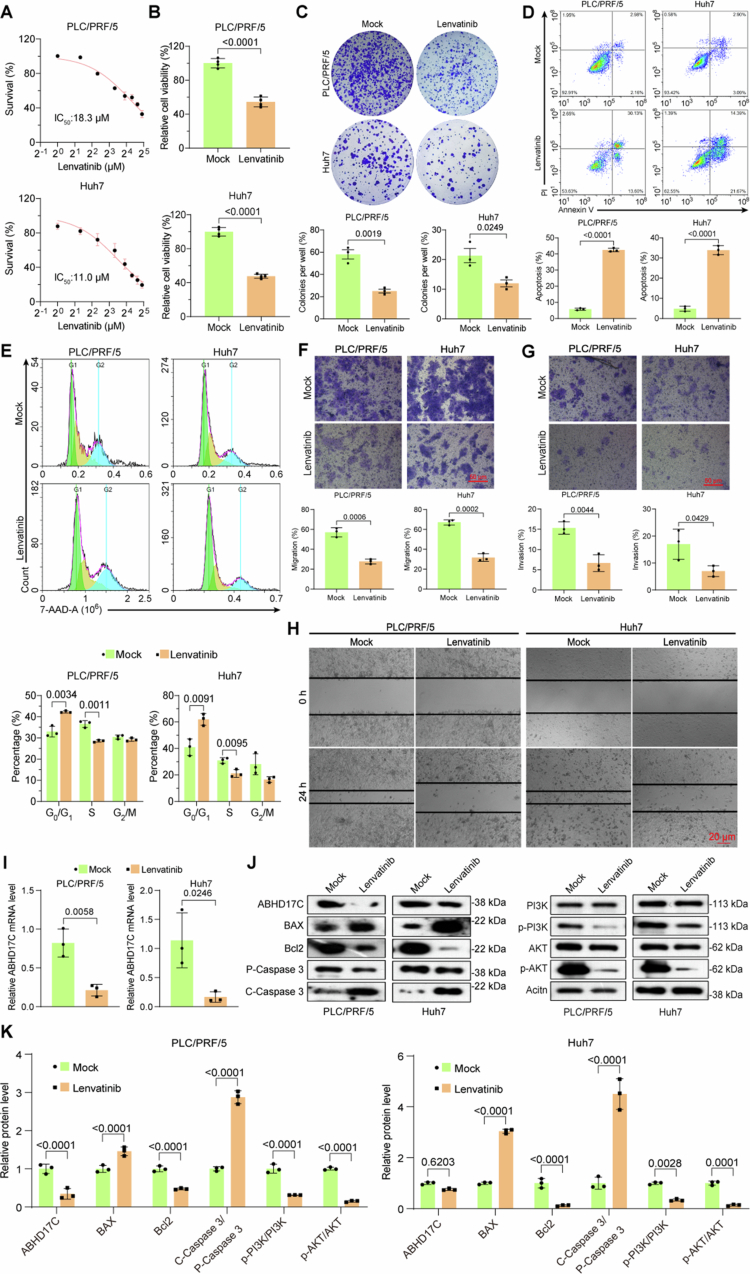
Lenvatinib decreases the viability, migration, invasion and induces apoptosis as well as affects the expression of ABHD17C and apoptosis-related proteins in HCC cells. (a) IC_50_ values of lenvatinib in PLC/PRF/5 and Huh7 cells determined by the MTT assay. The IC_50_ values were 18.3 µM for PLC/PRF/5 cells and 11.0 µM for Huh7 cells. (b) CCK-8 assay showing reduced cell viability in lenvatinib-treated HCC cells. (c–e) Colony formation (c), Annexin V-FITC/PI staining (d), and 7-AAD staining (e) demonstrating lenvatinib's effects on the colony formation capacity, apoptosis, and cell cycle progression of HCC cells. (f, g) Transwell data revealing the impact of lenvatinib on the migration (f) and invasion (g) of PLC/PRF/5 and Huh7 cells. (h) Wound healing results showing the reduced migration of lenvatinib-treated HCC cells. (i) qPCR data showing the expression of ABHD17C in control and lenvatinib-treated HCC cells. (j, k) Western blot analysis showing the expression of ABHD17C, BAX, Bcl2, pro-caspase 3 (P-caspase 3), cleaved caspase 3 (C-caspase 3), PI3K, p-PI3K, AKT, and p-AKT in control and lenvatinib-treated HCC cells. *p*-values were calculated by unpaired bilateral Student's *t*-test. Mock defines PBS treatment.

Single-cell transcriptomic analysis results imply that ABHD17C is linked not only to intrinsic tumor aggressiveness but also to a dysfunctional immune contexture that could limit effective anti-tumor responses. Based on this observation, we hypothesized that ABHD17C may also contribute to therapeutic resistance in HCC. Therefore, we next investigated the functional role of ABHD17C in modulating the anti-tumor effects of the multikinase inhibitor lenvatinib in HCC models. We first determined the IC_50_ values of lenvatinib in two HCC cell lines, PLC/PRF/5 and Huh7. Using the MTT assay, we found that the IC_50_ values were 18.3 µM for PLC/PRF/5 cells and 11.0 µM for Huh7 cells (Figure 2A). These concentrations were used in subsequent experiments. Next, a series of functional assays were conducted to evaluate the influence of lenvatinib on the malignant behavior of HCC cells. The CCK-8 assay confirmed that lenvatinib significantly reduced the viability of both PLC/PRF/5 and Huh7 cells (Figure 2B). Colony formation assays revealed a reduction in colony formation, while 7-AAD and Annexin V-FITC/PI staining demonstrated that lenvatinib induced cell cycle arrest and increased apoptosis in both cell lines (Figure 2C–E). Furthermore, Transwell and wound healing showed that lenvatinib markedly inhibited the migration and invasion capacities of PLC/PRF/5 and Huh7 cells (Figure 2F–H).

At the molecular level, qPCR and Western blot indicated that lenvatinib significantly downregulated ABHD17C mRNA and protein expression (Figure 2I–K). Additionally, Western blot revealed that lenvatinib reduced the expression of Bcl2 and the oncogenic signaling proteins p-PI3K and p-AKT, while upregulating BAX and cleaved Caspase 3 (Figure 2J and K). These findings confirm the anti-HCC effects of lenvatinib and its ability to suppress ABHD17C expression in HCC cells.

### ABHD17C overexpression mitigates the anti-tumor effect of lenvatinib in HCC cells

**Figure 3.**
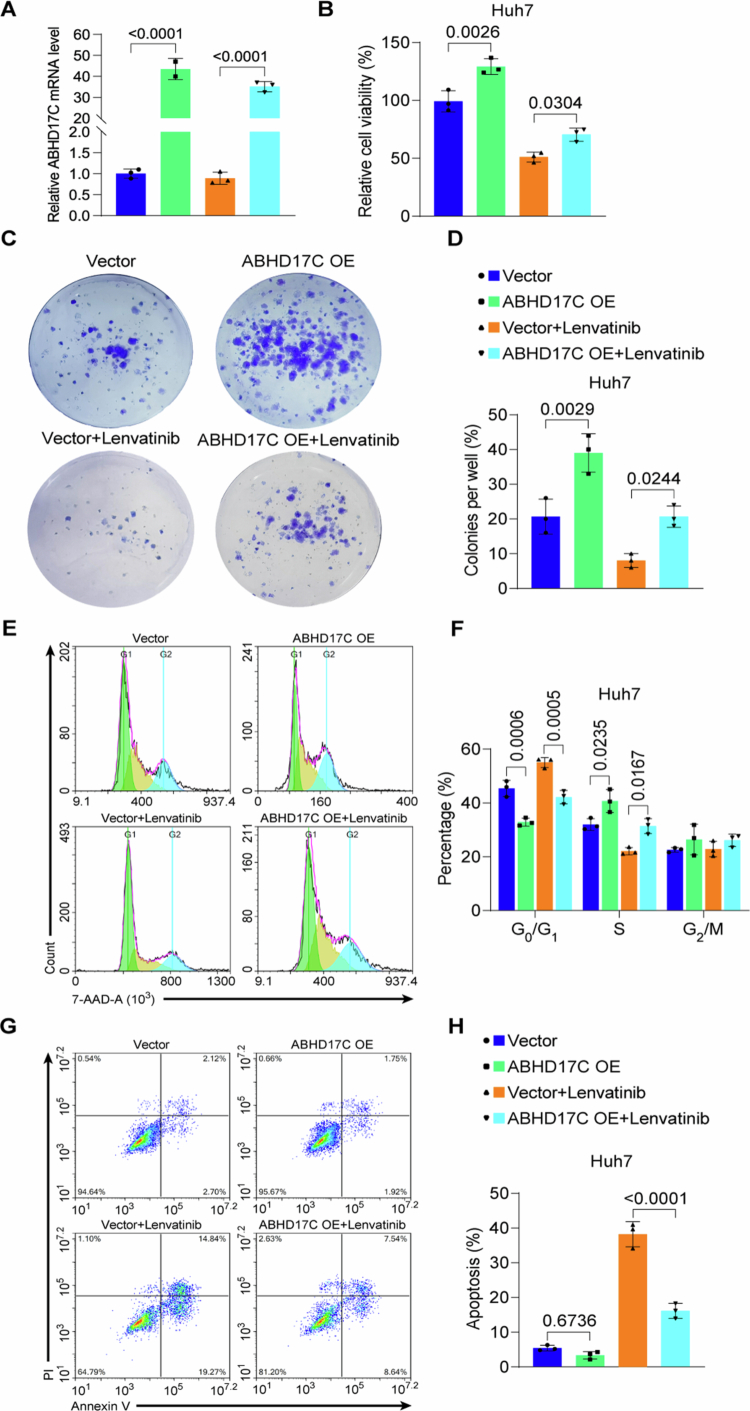
ABHD17C overexpression counteracts lenvatinib's anti-proliferative and pro-apoptotic effects in HCC cells. (a) qPCR data confirming ABHD17C overexpression in Huh7 cells. (b) CCK-8 results indicating the viability of Huh7 with the specified treatments. (c, d) Colony formation results showing the colony formation capacity of Huh7 with the specified treatments. (e, f) 7-AAD staining results depicting the cell cycle status of Huh7 with the specified treatments. (g, h) Annexin V-FITC/PI staining assay data illustrating apoptosis in Huh7 cells with the specified treatments. *p*-values were calculated by one-way ANOVA followed by Tukey's HSD.

**Figure 4.**
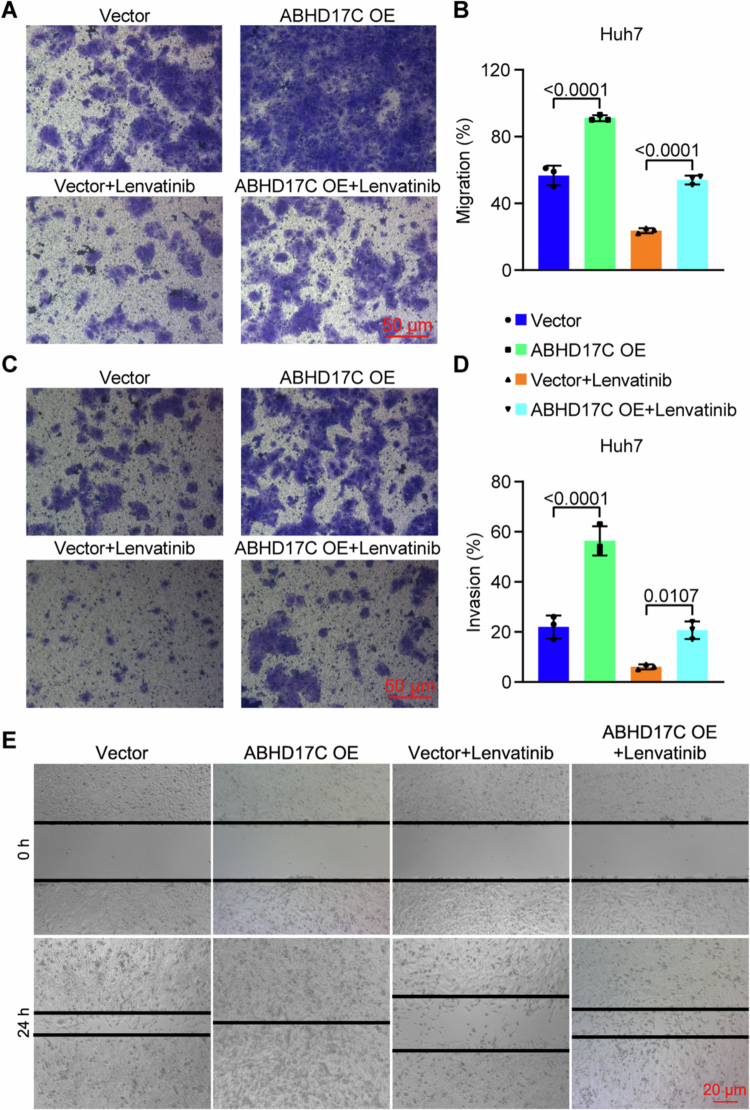
ABHD17C overexpression enhances the migration and invasion in HCC cells. (a–d) Transwell results demonstrating the migration (a, b) and invasion (c, d) of Huh7 with the specified treatments. *p*-values were calculated by one-way ANOVA followed by Tukey's HSD. (e) Wound healing data showing the migration of Huh7 with the specified treatments.

**Figure 5.**
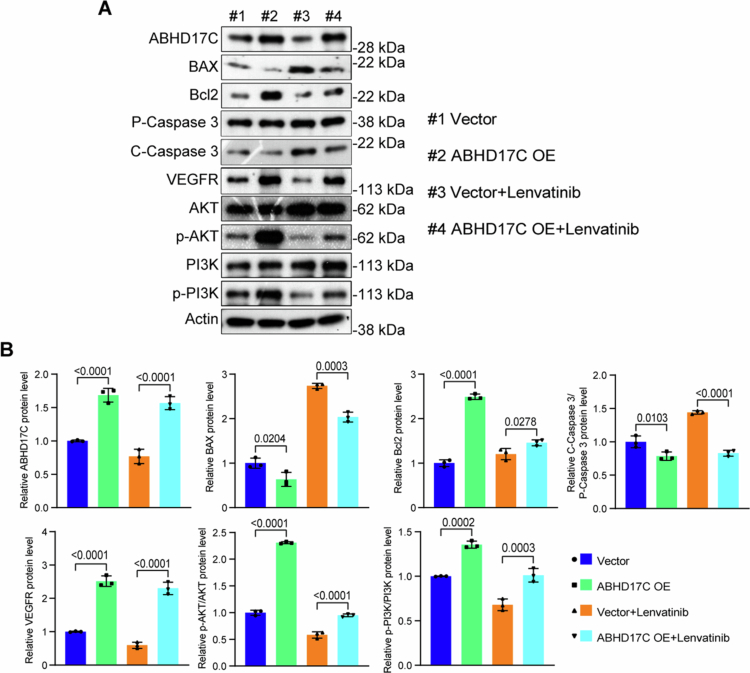
ABHD17C overexpression modulates apoptosis regulators and the PI3K/AKT pathway in HCC cells. (a, b) Western blot showing the expression of ABHD17C, BAX, Bcl2, pro-caspase 3 (P-caspase 3), cleaved caspase 3 (C-caspase 3), VEGFR, PI3K, p-PI3K, AKT, and p-AKT in Huh7 cells with the specified treatments. *p*-values were calculated by one-way ANOVA followed by Tukey's HSD.

Next, we overexpressed ABHD17C in Huh7 and treated them with lenvatinib. The efficiency of ABHD17C overexpression was confirmed by qPCR (Figure 3A). CCK-8 assay, colony formation, 7-AAD staining, and Annexin V-FITC/PI staining revealed that ABHD17C overexpression significantly increased cell proliferation, enhanced colony formation, promoted cell cycle progression, and reduced apoptosis in Huh7 cells, regardless of lenvatinib treatment (Figure 3B–H). Similarly, Transwell and wound healing demonstrated that ABHD17C overexpression enhanced the migration and invasion capacities of both untreated and lenvatinib-treated Huh7 cells (Figure 4A–E). Additionally, Western blot showed that ABHD17C overexpression increased the levels of Bcl2, VEGFR, p-AKT, and p-PI3K while suppressing the expression of BAX and cleaved Caspase 3 in both lenvatinib-treated and untreated Huh7 cells (Figure 5A and B).

Consistently, molecular docking analysis indicated a potential interaction between ABHD17C and lenvatinib, with a binding score of −7.9 kcal/mol, suggesting moderate binding affinity (Figure S3). These findings suggest that ABHD17C counteracts the tumor-suppressive effects of lenvatinib in HCC cells.

### ABHD17C depletion enhances the anti-tumor effect of lenvatinib in HCC cells

**Figure 6.**
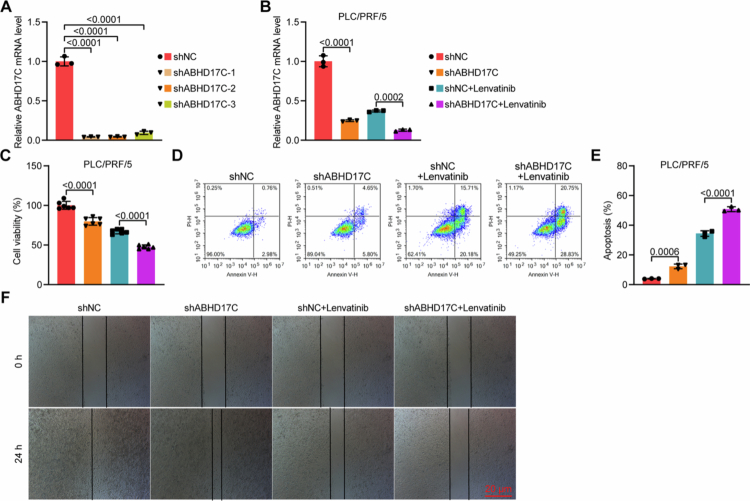
ABHD17C knockdown enhances the anti-tumor effects of lenvatinib in HCC cells. (a) qPCR validation of ABHD17C knockdown efficiency in HEK293T cells using three independent shRNAs. shABHD17C-1 showing the highest knockdown efficiency. (b) qPCR confirmation of ABHD17C silencing efficiency in PLC/PRF/5 cells following shRNA transduction with/without lenvatinib treatment. (c) CCK-8 assay showing that ABHD17C knockdown further reduces cell viability in lenvatinib-treated PLC/PRF/5 cells. (d, e) Annexin V-FITC/PI staining demonstrating that ABHD17C depletion enhances lenvatinib-induced apoptosis. (f) Wound healing assay showing that ABHD17C knockdown suppresses the migration in lenvatinib-treated cells. *p*-values were calculated by one-way ANOVA followed by Tukey's HSD.

To further validate the findings obtained from ABHD17C overexpression experiments, ABHD17C was silenced using specific shRNAs. qPCR analysis confirmed that all three shABHD17C constructs effectively reduced ABHD17C expression in HEK293T cells, with shABHD17C-1 showing the highest knockdown efficiency and therefore selected for subsequent experiments (Figure 6A). Efficient ABHD17C silencing in PLC/PRF/5 cells was further confirmed by qPCR following shABHD17C transduction (Figure 6B).

**Figure 7.**
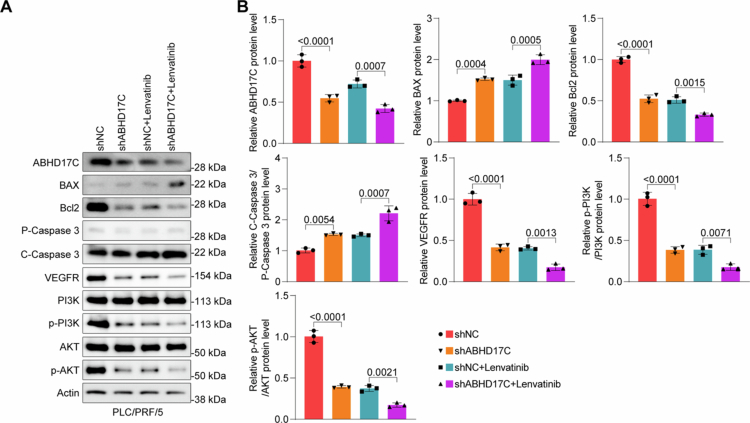
ABHD17C knockdown enhances lenvatinib-induced apoptosis and suppresses oncogenic signaling in HCC cells. (a, b) Western blot analysis showing that ABHD17C depletion decreases Bcl2, VEGFR, p-PI3K, and p-AKT expression, while increasing BAX and cleaved caspase-3 levels in lenvatinib-treated PLC/PRF/5 cells. *p*-values were calculated by one-way ANOVA followed by Tukey's HSD.

Functional assays demonstrated that ABHD17C knockdown significantly enhanced the anti-tumor effects of lenvatinib in PLC/PRF/5 cells. Specifically, ABHD17C depletion further reduced cell viability, promoted apoptosis, and impaired the migratory capacity of lenvatinib-treated cells, as shown by CCK-8, Annexin V-FITC/PI staining, and wound healing assays (Figure 6C–F). Consistently, Western blot analysis revealed that ABHD17C depletion decreased the expression of Bcl2, VEGFR, p-AKT, and p-PI3K, while increasing the levels of BAX and cleaved Caspase 3 in lenvatinib-treated PLC/PRF/5 cells (Figure 7A and B). Collectively, these findings further support that ABHD17C attenuates the anti-tumor efficacy of lenvatinib in HCC cells.

### Excessive ABHD17C attenuates the anti-tumor effect of lenvatinib in an HCC xenograft mouse model

**Figure 8.**
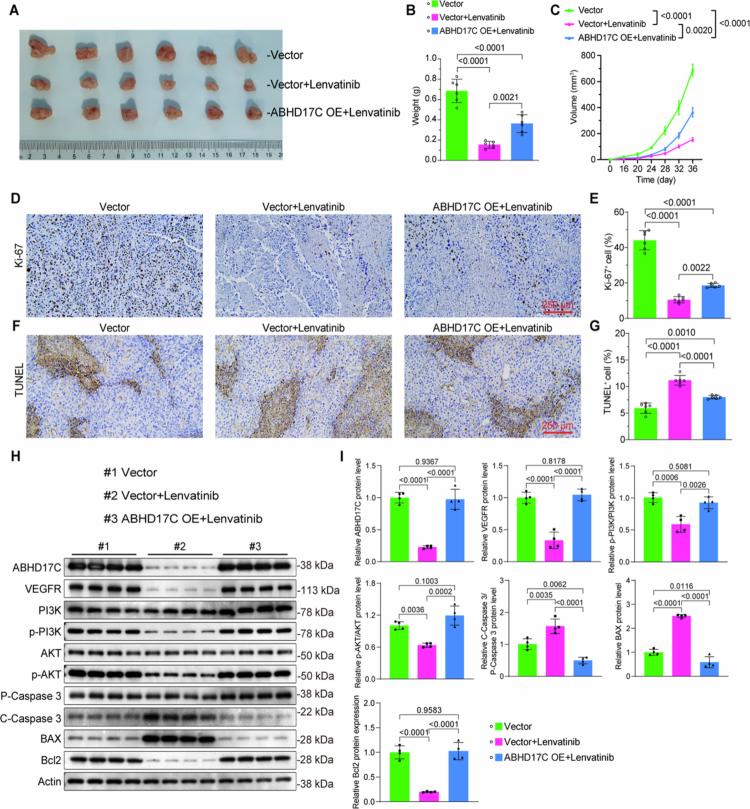
ABHD17C overexpression attenuates lenvatinib's anti-tumor effects in an HCC xenograft model. (a) Overall morphology of xenograft tumors derived from Huh7 cells with the indicated treatments. (b) Tumor weight on Day 36. (c) Volume of tumors during a 36-d time course. (d, e) IHC data showing the expression of Ki-67 in the tumors of each group. (f, g) TUNEL assay results demonstrating apoptosis in the tumors of each group. (h, i) Western blot analysis showing the expression of ABHD17C, BAX, Bcl2, pro-caspase 3 (P-caspase 3), cleaved caspase 3 (C-caspase 3), VEGFR, PI3K, p-PI3K, AKT, and p-AKT in tumors with the indicated treatments. One-way ANOVA followed by Tukey's HSD test was applied to calculate *p*-values.

To validate these in vitro findings, we employed an HCC xenograft mouse model. After 36 d of development, tumors derived from control HCC cells showed a significant reduction in size upon lenvatinib treatment; however, this effect was diminished in tumors derived from ABHD17C-overexpressing cells (Figure 8A–C). IHC analysis revealed that lenvatinib reduced the percentage of Ki-67^+^ proliferative cells in control tumors, but this anti-proliferative effect was attenuated in ABHD17C-overexpressing tumors (Figure 8D and E). Similarly, the pro-apoptotic effect of lenvatinib was compromised in tumors with ABHD17C overexpression (Figure 8F and G).

Western blot analysis of tumor tissues demonstrated that lenvatinib treatment downregulated ABHD17C and VEGFR expression in control tumors while reducing the levels of p-PI3K, p-AKT, and Bcl2 and increasing cleaved Caspase 3 and BAX expression. In contrast, these effects were abrogated in tumors derived from ABHD17C-overexpressing cells (Figure 8H and I). These in vivo results confirm that ABHD17C overexpression diminishes the anti-tumor effect of lenvatinib in HCC.

### ABHD17C overexpression weakens the lenvatinib's efficacy in HCC organoids

**Figure 9.**
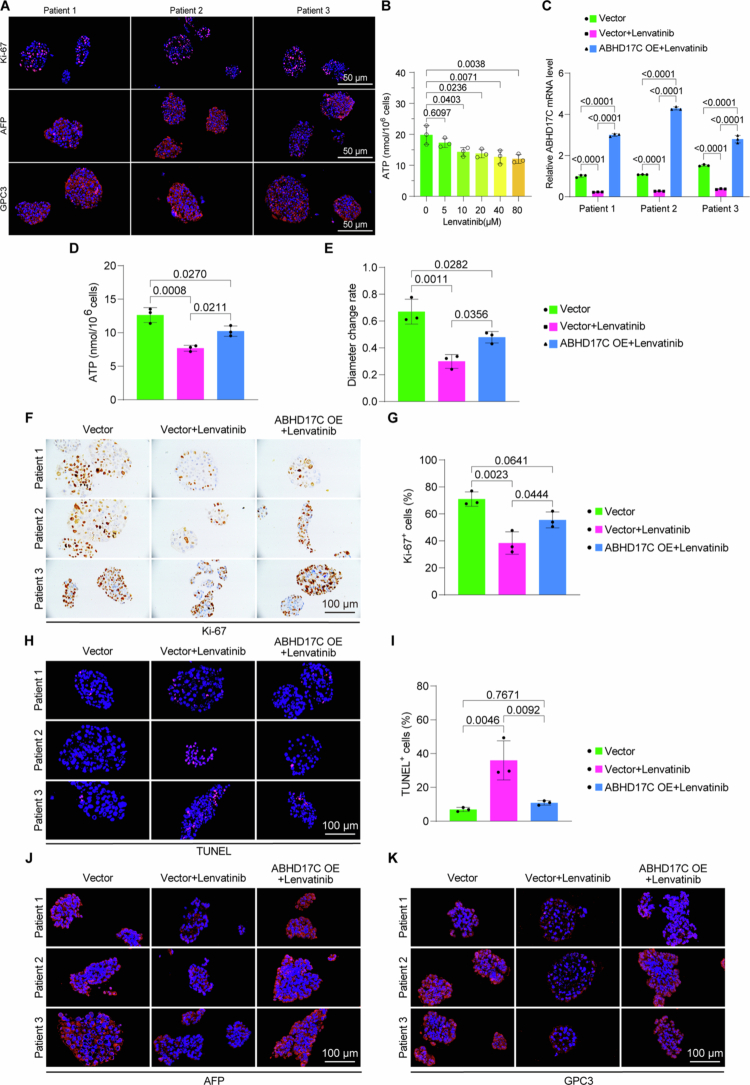
ABHD17C overexpression diminishes lenvatinib's efficacy in HCC organoids. (a) IF data showing the expression of Ki-67, AFP, and GPC3 in the HCC organoids. (b) ATP assay showing the viability of HCC organoids exposed to graded concentrations of lenvatinib. (c) qPCR data indicating ABHD17C mRNA expression in organoids with the specified treatments. (d) ATP assay data depicting the viability of the organoids in each group. (e) The ratio of organoid diameter before and after lenvatinib treatment. (f, g) IHC data demonstrating the presence of Ki-67^+^ cells in HCC organoids with the indicated treatments. (h, i) TUNEL assay data illustrating the apoptosis in the HCC organoids in each group. (j, k) IF results showing the expression of the HCC markers AFP (j) and GPC3 (k) in HCC organoids with the indicated treatments. For *p*-value calculation, two-way ANOVA followed by Tukey's multiple comparison test was used for the data in (c), and one-way ANOVA followed by Tukey's HSD test was used for the data in (b–i).

To further explore the function of ABHD17C in lenvatinib sensitivity, we employed organoids derived from primary HCC tissues obtained from three independent patients. IF staining confirmed that these organoids were viable and expressed the HCC markers AFP and GPC3 (Figure 9A). Next, we optimized the lenvatinib concentration for HCC organoids with ATP assay, which revealed that concentrations ≥ 10 µM significantly inhibited organoid viability (Figure 9B). Thus, 10 µM lenvatinib was selected for subsequent experiments.

We then overexpressed ABHD17C in these organoids and verified its overexpression efficacy by qPCR. Notably, lenvatinib treatment significantly suppressed ABHD17C expression in HCC organoids (Figure 9C). Functional assays demonstrated that ABHD17C overexpression partially rescued the reduction in organoid viability induced by lenvatinib, as shown by ATP assays (Figure 9D). Consistently, the diameter of the lenvatinib-treated organoids increased with ABHD17C overexpression, and the percentage of Ki-67^+^ proliferative cells was elevated in the ABHD17C-overexpressing organoids (Figure 9E‒G). TUNEL assays revealed that the pro-apoptotic effect of lenvatinib was abolished in ABHD17C-overexpressing organoids (Figure 9H and I). IF staining further showed that lenvatinib dramatically reduced the expression of the HCC markers AFP and GPC3 in control organoids, but this effect was attenuated in ABHD17C-overexpressing organoids (Figure 9J and K).

**Figure 10.**
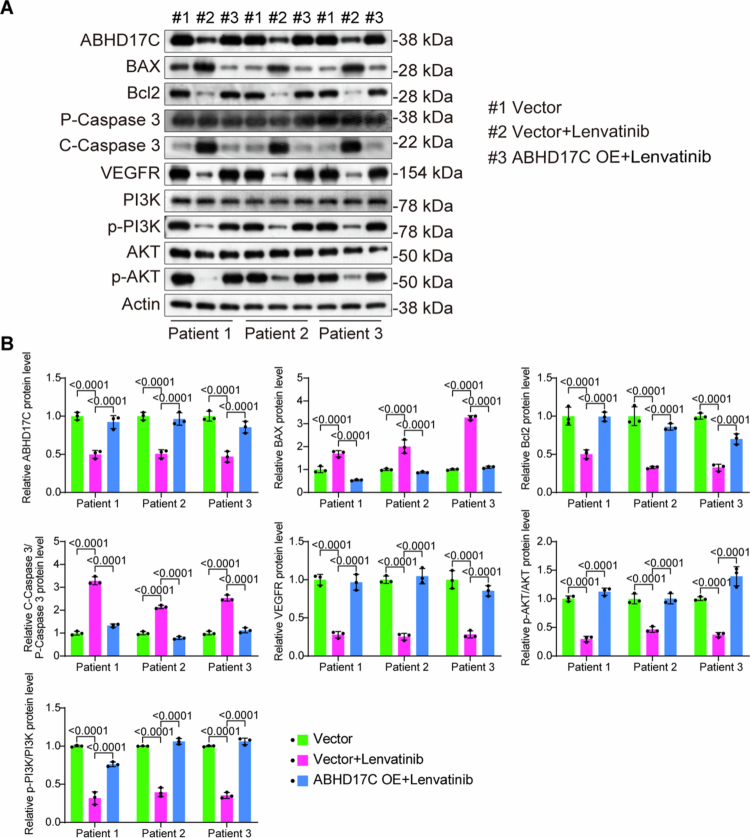
ABHD17C overexpression affects apoptosis regulators and the PI3K/AKT pathway in HCC organoids. (a, b) Western blot revealing the expression of ABHD17C, BAX, Bcl2, pro-caspase 3 (P-caspase 3), cleaved caspase 3 (C-Caspase 3), VEGFR, PI3K, p-PI3K, AKT, and p-AKT in HCC organoids with the indicated treatments. Two-way ANOVA followed by Tukey's multiple comparison test was applied to calculate *p*-values.

At the molecular level, Western blot analysis demonstrated that lenvatinib downregulated ABHD17C, VEGFR, p-PI3K, p-AKT, and Bcl2 while upregulating cleaved Caspase 3 and BAX in control organoids. However, these effects were reversed in ABHD17C-overexpressing organoids (Figure 10A and B). Together, these findings provide further evidence that ABHD17C overexpression disrupts the anti-tumor effect of lenvatinib in HCC.

## Discussion

Like most anti-cancer therapies, lenvatinib treatment in HCC is frequently limited by the emergence of drug resistance, underscoring the need to elucidate the mechanisms underlying reduced therapeutic responsiveness. In this study, we identified ABHD17C as a potential regulator linking tumor progression, immune suppression, and lenvatinib sensitivity in HCC. Our scRNA-seq analysis revealed that high ABHD17C expression was associated with an immunosuppressive tumor microenvironment characterized by reduced infiltration of CD8⁺ T cells and NK cells, expansion of exhausted T-cell populations, and impaired intercellular communication. Notably, key anti-tumor signaling pathways, including CXCL, LCK/TCR, IFN-II, and CCL signaling, were diminished, whereas immune evasion–associated interactions, such as CD96–PVR and ADGRE5–CD55, were enhanced, suggesting that ABHD17C may contribute to immune escape and dysfunctional anti-tumor immunity in HCC.

Although ABHD17C was relatively enriched across hepatocytes, myeloid cells, and T/NK cells, no significant difference in expression was observed between malignant and non-malignant hepatocytes. This suggests that its pathological role may be more closely related to shaping the tumor microenvironment than to direct hepatocyte malignant transformation. However, given that ABHD17C is overexpressed in HCC cells in bulk analyses, this apparent discrepancy may reflect limitations of scRNA-seq sensitivity for low-to-moderately expressed transcripts, sample size constraints, and potential uncertainties in tumor cell annotation based on CNV inference. Importantly, the pronounced enrichment of exhausted CD8⁺ T cells in ABHD17C-high tumors further supports a role for ABHD17C in promoting T-cell dysfunction. As effective CD8⁺ T-cell activity is essential for tumor immune surveillance and therapeutic response, these findings provide a mechanistic context for the reduced efficacy of lenvatinib observed in ABHD17C-overexpressing models.

ABHD17C is overexpressed in HCC, but whether its expression levels differ between lenvatinib-resistant and lenvatinib-responsive patients remains unclear. Our previous studies have identified mechanisms regulating ABHD17C expression in HCC, such as miR-145-5p-mediated mRNA regulation and USP35-mediated protein stabilization.^18^ ^,^ ^21^ Given that lenvatinib significantly suppresses ABHD17C expression in HCC cells and organoids, it would be valuable to investigate whether lenvatinib modulates ABHD17C through these mechanisms.

Lenvatinib has been shown to regulate key malignant behaviors in HCC, including proliferation, cell cycle progression, apoptosis, migration, and invasion. Similarly, ABHD17C has been implicated in modulating these processes,^18^ which explains why its overexpression counteracts lenvatinib's effects. Notably, our recent work also highlights ABHD17C’s role in suppressing pyroptosis in HCC cells.^19^ Pyroptosis is a type of programmed cell death, usually triggered by infection or injury, which is characterized by the rupture of cell membranes, the release of inflammatory factors, and the triggering of a strong inflammatory response. Since targeting pyroptosis is a potential therapeutic strategy for HCC,^22^ and lenvatinib can affect pyroptosis in HCC,^23^ it would be intriguing to explore whether ABHD17C interferes with lenvatinib's regulation of pyroptosis in HCC, which contributes to our deeper understanding of the molecular mechanism of lenvatinib in HCC.

While our studies focused on HCC, ABHD17C's role in other cancers is also emerging. For instance, ABHD17C has prognostic significance in pancreatic cancer, where it may act as an oncogene.^24^ ^,^ ^25^ However, its functions in other cancers remain poorly understood. Given that ABHD17C regulates the NRAS pathway, ABHD17C regulates the membrane localization of Ras proteins by depalmitoylation and may affect the activity of the Ras signaling pathway,^15^ which is frequently dysregulated in various cancers,^26^ it is plausible that ABHD17C plays a broader role in tumorigenesis. In addition, ABHD17C may affect tumor angiogenesis by regulating the activity of VEGFR and other signaling molecules. Moreover, ABHD17C may affect the immune escape, migration and invasion of tumor cells by regulating the activity of immune-related proteins and cytoskeleton-related proteins. Since lenvatinib is used to treat multiple cancers beyond HCC, further research is needed to determine whether ABHD17C 'ediates lenvatinib sensitivity in these contexts.

Although this study reveals the important role of ABHD17C in mediating lenvatinib sensitivity in HCC, several limitations should be acknowledged. First, the scRNA-seq findings were derived from a single public dataset and require validation in larger independent patient cohorts, as well as further functional studies to confirm the mechanistic role of ABHD17C in shaping the tumor microenvironment. Second, the BALB/c nude mouse model used in this study cannot fully recapitulate the complete immune microenvironment of HCC patients, which limits the clinical translatability of our findings and the ability to fully assess the role of ABHD17C in remodeling the tumor microenvironment. Third, the potential interaction between ABHD17C and lenvatinib was supported only by molecular docking analysis and thus requires further biochemical and biophysical validation to confirm direct binding and its functional relevance in HCC. Finally, the concentrations of lenvatinib used in the in vitro and organoid experiments, while suitable for mechanistic investigations, may not fully reflect physiologically achievable or clinically relevant drug exposures in patients; therefore, the translational relevance of the observed sensitivity-related mechanisms should be interpreted with caution.

In summary, our study reveals that ABHD17C overexpression diminishes the anti-tumor efficacy of lenvatinib in HCC, providing new insights into the mechanisms of lenvatinib sensitivity. These findings highlight ABHD17C as a potential therapeutic target to enhance drug sensitivity and improve HCC treatment outcomes.

## Supplementary Material

Supplementary material Figure S2.tif

Figure S1.tif

Supplementary material Figure S3.jpg

Supplementary material ARRIVE Checklist

Supplementary material Supplementary figures caption

## Data Availability

The data that support the findings of this study are openly available in Figshare at https://doi.org/10.6084/m9.figshare.32706510. The data involved human subjects are not publicly available due to the containing information that could compromise the privacy of research participants.

## References

[1] Schlumberger M , Tahara M , Wirth LJ , Robinson B , Brose MS , Elisei R , Habra MA , Newbold K , Shah MH , Hoff AO , et al. Lenvatinib versus placebo in radioiodine-refractory thyroid cancer. NEJM. 2015;372(7):621–630. doi: 10.1056/NEJMoa1406470. 25671254

[2] Chen Y , Dai S , Cheng CS , Chen L . Lenvatinib and immune-checkpoint inhibitors in hepatocellular carcinoma: mechanistic insights, clinical efficacy, and future perspectives. J Hematol Oncol. 2024;17(1):130. doi: 10.1186/s13045-024-01647-1. 39709431 PMC11663365

[3] Santoni M , Mollica V , Rizzo A , Massari F . Dynamics of resistance to immunotherapy and TKI in patients with advanced renal cell carcinoma. Cancer Treat Rev. 2025;133:102881. doi: 10.1016/j.ctrv.2025.102881. 39799795

[4] Chen P , Yao Y , Tan H , Li J . Systemic treatments for radioiodine-refractory thyroid cancers. Front Endocrinol (Lausanne). 2024;15:1346476. doi: 10.3389/fendo.2024.1346476. 39473507 PMC11518755

[5] Li J , Yang B , Teng Z , Liu Y , Li D , Qu X . Efficacy and safety of first-line treatments for advanced hepatocellular carcinoma patients: a systematic review and network meta-analysis. Front Immunol. 2024;15:1430196. doi: 10.3389/fimmu.2024.1430196. 39355238 PMC11442238

[6] Ye G , Ye M . Jin X. Roles of clinical application of lenvatinib and its resistance mechanism in advanced hepatocellular carcinoma (Review). Am J Cancer Res. 2024;14(9):4113–4171. doi: 10.62347/ujvp4361. 39417171 PMC11477829

[7] You Q , Li R , Yao J , Zhang YC , Sui X , Xiao CC , Chen H , Zheng J , Yang Y . Insights into lenvatinib resistance: mechanisms, potential biomarkers, and strategies to enhance sensitivity. Med Oncol. 2024;41(3):75. doi: 10.1007/s12032-023-02295-0. 38381181

[8] Oura K , Morishita A , Hamaya S , Fujita K , Masaki T . The roles of epigenetic regulation and the tumor microenvironment in the mechanism of resistance to systemic therapy in hepatocellular carcinoma. Int J Mol Sci. 2023;24(3):2805. doi: 10.3390/ijms24032805. 36769116 PMC9917861

[9] Sobocinska J , Roszczenko-Jasinska P , Ciesielska A , Kwiatkowska K . Protein palmitoylation and its role in bacterial and viral infections. Front Immunol. 2017;8:2003. doi: 10.3389/fimmu.2017.02003. 29403483 PMC5780409

[10] Rocks O , Gerauer M , Vartak N , Koch S , Huang ZP , Pechlivanis M , Kuhlmann J , Brunsveld L , Chandra A , Ellinger B , et al. The palmitoylation machinery is a spatially organizing system for peripheral membrane proteins. Cell. 2010;141(3):458–471. doi: 10.1016/j.cell.2010.04.007. 20416930

[11] Won SJ , Martin BR . Temporal profiling establishes a dynamic S-Palmitoylation cycle. ACS Chem Biol. 2018;13(6):1560–1568. doi: 10.1021/acschembio.8b00157. 29733200 PMC6192522

[12] Martin BR , Wang C , Adibekian A , Tully SE , Cravatt BF . Global profiling of dynamic protein palmitoylation. Nat Methods. 2011;9(1):84–89. doi: 10.1038/nmeth.1769. 22056678 PMC3248616

[13] Main A , Fuller W . Protein S-Palmitoylation: advances and challenges in studying a therapeutically important lipid modification. FEBS J. 2022;289(4):861–882. doi: 10.1111/febs.15781. 33624421

[14] Lin DT , Conibear E . ABHD17 proteins are novel protein depalmitoylases that regulate N-Ras palmitate turnover and subcellular localization. eLife. 2015;4:e11306. doi: 10.7554/eLife.11306. 26701913 PMC4755737

[15] Remsberg JR , Suciu RM , Zambetti NA , Hanigan TW , Firestone AJ , Inguva A , Long A , Ngo N , Lum KM , Henry CL , et al. ABHD17 regulation of plasma membrane palmitoylation and N-Ras-dependent cancer growth. Nat Chem Biol. 2021;17(8):856–864. doi: 10.1038/s41589-021-00785-8. 33927411 PMC8900659

[16] Zhou B , Hao Q , Liang Y , Kong E . Protein palmitoylation in cancer: molecular functions and therapeutic potential. Mol Oncol. 2023;17(1):3–26. doi: 10.1002/1878-0261.13308. 36018061 PMC9812842

[17] Ko PJ , Dixon SJ . Protein palmitoylation and cancer. EMBO Rep. 2018;19(10):e46666. doi: 10.15252/embr.201846666. 30232163 PMC6172454

[18] Wang L , Wang J , Ma X , Ju G , Shi C , Wang W , Wu J . USP35 promotes HCC development by stabilizing ABHD17C and activating the PI3K/AKT signaling pathway. Cell Death Discov. 2023;9(1):421. doi: 10.1038/s41420-023-01714-5. 37993419 PMC10665393

[19] Wang L , Wang J , Shi C , Wang W , Wu J . ABHD17C represses apoptosis and pyroptosis in hepatocellular carcinoma cells. Biocell. 2024;48(9):1299–1310. doi: 10.32604/biocell.2024.051756.

[20] Broutier L , Mastrogiovanni G , Verstegen MM , Francies HE , Gavarró LM , Bradshaw CR , Allen GE , Arnes-Benito R , Sidorova O , Gaspersz MP , et al. Human primary liver cancer-derived organoid cultures for disease modeling and drug screening. Nat Med. 2017;23(12):1424–1435. doi: 10.1038/nm.4438. 29131160 PMC5722201

[21] Wang L , Ma X , Chen Y , Zhang J , Zhang J , Wang W , Chen S . MiR-145-5p suppresses hepatocellular carcinoma progression by targeting ABHD17C. Oncologie. 2022;24(4):897–912. doi: 10.32604/oncologie.2022.025693.

[22] Liu RJ , Yu XD , Yan SS , Guo ZW , Zao XB , Zhang YS . Ferroptosis, pyroptosis and necroptosis in hepatocellular carcinoma immunotherapy: mechanisms and immunologic landscape (Review). Int J Oncol. 2024;64(6):63. doi: 10.3892/ijo.2024.5651. 38757345 PMC11095606

[23] Fu XW , Song CQ . Identification and validation of pyroptosis-related gene signature to predict prognosis and reveal immune infiltration in hepatocellular carcinoma. Front Cell Dev Biol. 2021;9:748039. doi: 10.3389/fcell.2021.748039. 34820376 PMC8606409

[24] Zhang W , Xie Y , Yu X , Liu C , Gao W , Xing W , Si T . ABHD17C, a metabolic and immune-related gene signature, predicts prognosis and anti-PD1 therapy response in pancreatic cancer. Discov Oncol. 2023;14(1):87. doi: 10.1007/s12672-023-00690-7. 37273016 PMC10241759

[25] Chen Z , Du Y , Shi H , Dong S , He R , Zhou W . Long non-coding RNA MIR4435-2HG promotes pancreatic cancer progression by regulating ABHD17C through sponging miR-128-3p. Transl Cancer Res. 2024;13(8):4113–4130. doi: 10.21037/tcr-24-51. 39262472 PMC11385540

[26] Yang X , Wu H . RAS signaling in carcinogenesis, cancer therapy and resistance mechanisms. J Hematol Oncol. 2024;17(1):108. doi: 10.1186/s13045-024-01631-9. 39522047 PMC11550559

