# Supplementary figures and images for: ABHD17C regulates the efficacy of lenvatinib in suppressing hepatocellular carcinoma
Source: Cancer Biol Ther. 2026 Jun 25;27(1):2693350. doi: 10.1080/15384047.2026.2693350 (PMC13313231; doi:10.1080/15384047.2026.2693350)

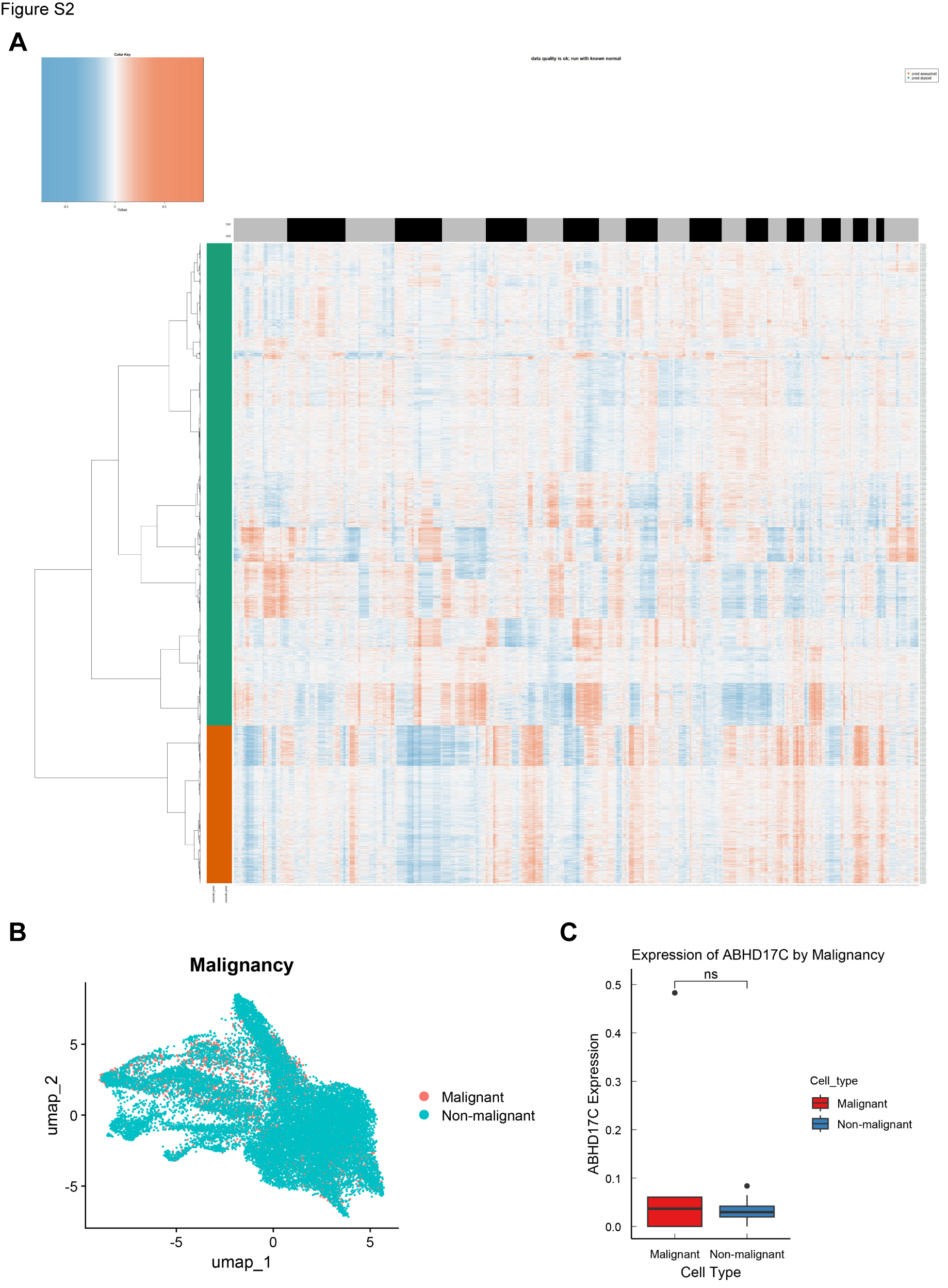

Supplement: Supplementary material — Figure S2.tif [file KCBT_A_2693350_SM0307.tif]

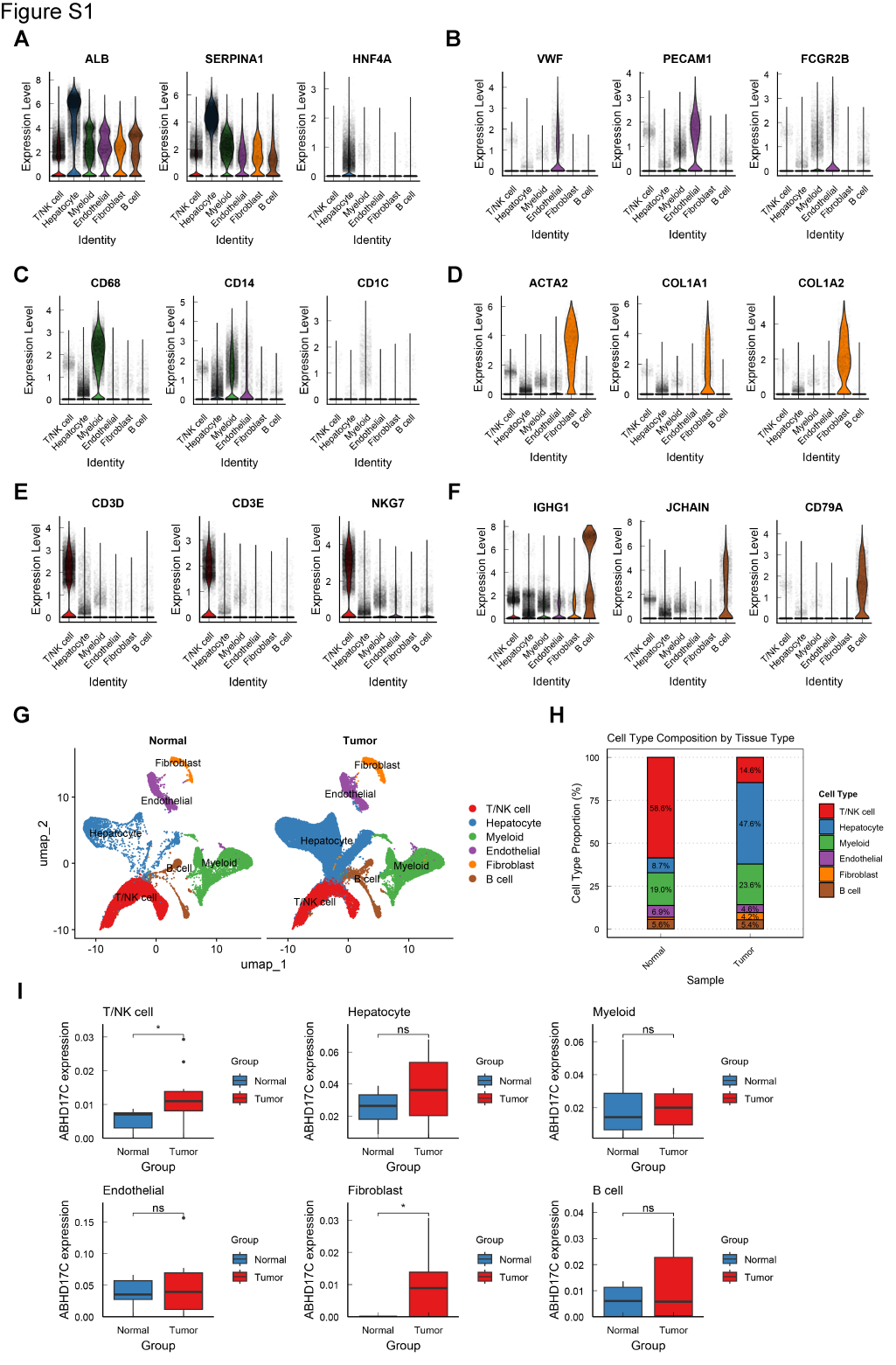

Supplement: Figure S1.tif [file KCBT_A_2693350_SM0309.tif]

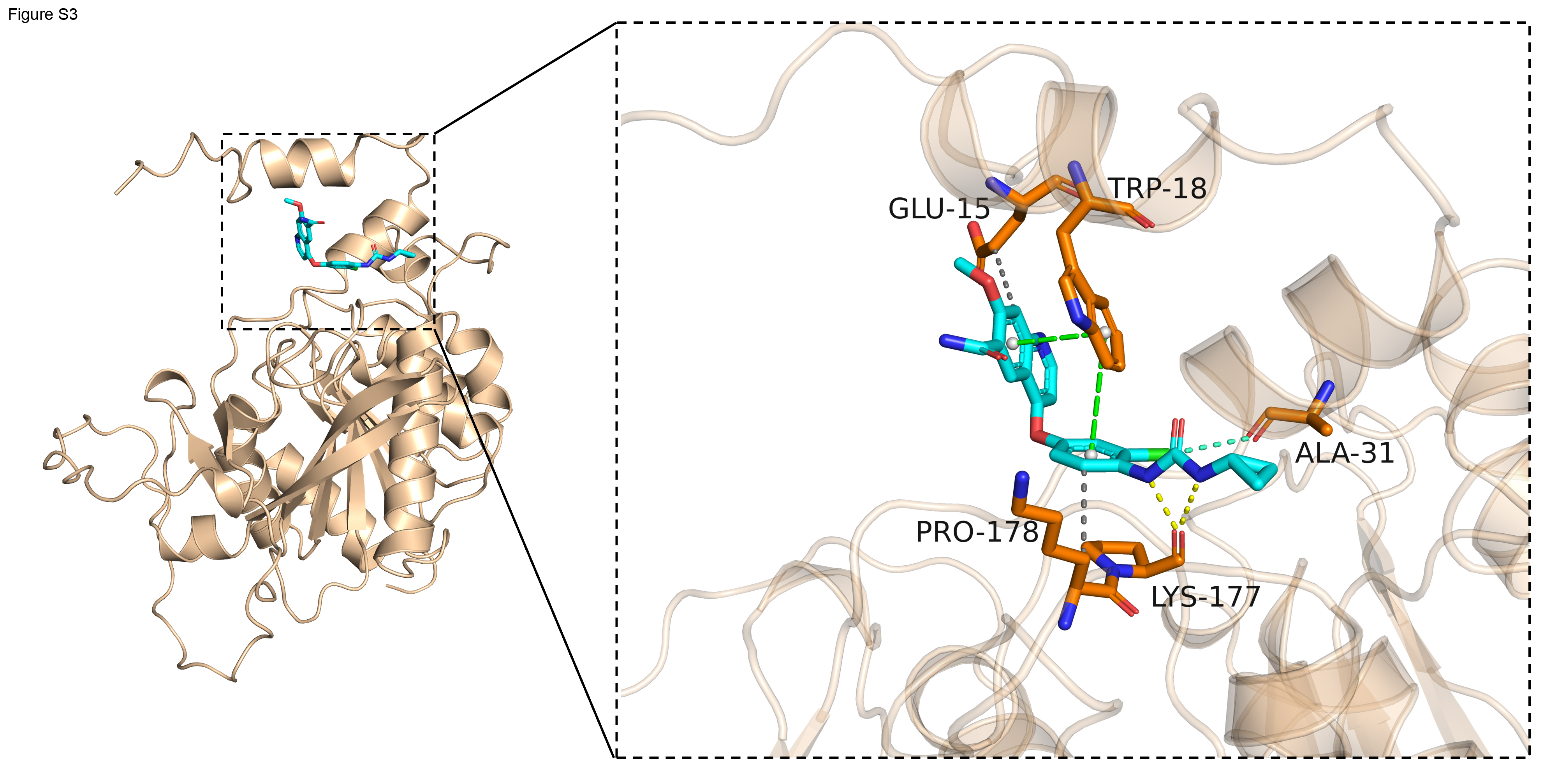

Supplement: Supplementary material — Figure S3.jpg [file KCBT_A_2693350_SM0308.jpg]
