# Supplementary material for: ABHD17C regulates the efficacy of lenvatinib in suppressing hepatocellular carcinoma
Source: Cancer Biol Ther. 2026 Jun 25;27(1):2693350. doi: 10.1080/15384047.2026.2693350 (PMC13313231; doi:10.1080/15384047.2026.2693350)
Supplement: Supplementary material — Supplementary figures caption [file KCBT_A_2693350_SM2564.docx]

**Figure S1. Annotation and global cellular landscape of the HCC.** (A–F) Expression of canonical marker genes used for cell type annotation, including CD3D/CD3E/NKG7 (T/NK cells), IGHG1/JCHAIN/CD79A (B cells), CD68/CD14/CD1C (myeloid cells), ALB/SERPINA1/HNF4A (hepatocytes), VWF/PECAM1/FCGR2B (endothelial cells), and ACTA2/COL1A1/COL1A2 (fibroblasts). (G) Overall distribution of major cell populations across tumor and adjacent normal tissues. (H) Relative proportions of major cell types. (I) Differential expression analysis of ABHD17C across cell types between tumor and normal tissues. *, *p*<0.05; ns, not significant. Wilcoxon Rank Sum Test is used for statistical analysis of (I).

**Figure S2. Assessment of hepatocyte malignancy and ABHD17C expression in malignant versus non-malignant hepatocytes.** (A) CopyKAT-inferred CNV heatmap used to distinguish malignant and non-malignant hepatocytes. (B) UMAP visualization of hepatocyte populations classified as malignant or non-malignant based on CNV profiles. (C) Comparison of ABHD17C expression levels between malignant and non-malignant hepatocytes. ns, not significant. Wilcoxon Rank Sum Test is used for statistical analysis of (C).

**Figure S3. Molecular docking analysis of ABHD17C and Lenvatinib.** Molecular docking analysis was performed to predict the binding interaction between ABHD17C and lenvatinib. The left panel shows the overall binding conformation, while the right panel illustrates the local interaction details. In the figure, cyan sticks represent lenvatinib, and orange cartoons represent ABHD17C. Yellow dashed lines indicate hydrogen bonds, gray dashed lines represent hydrophobic interactions, and cyan dashed lines represent halogen bonds. Docking simulations were conducted using AutoDock Vina (v1.1.2), and structural visualization was generated using PyMOL (v2.5.2).
